# Gene expression analysis of primary graft dysfunction in adult heart transplant recipients: A cohort study

**DOI:** 10.1016/j.jhlto.2025.100215

**Published:** 2025-01-22

**Authors:** Samuel Padovani Steffen, Alvaro Monteiro Perazzo, Shirlyne Fabianni Dias Gaspar, Ronaldo Honorato Barros Santos, Domingos Dias Lourenço Filho, Alexandre da Costa Pereira, Luis Fernando B. da Costa Seguro, Fabiana G. Marcondes-Braga, Fernando Bacal, Jose Eduardo Krieger, Fabio Antonio Gaiotto, Fabio Biscegli Jatene

**Affiliations:** a Heart Transplant Unit, Heart Institute of Clinical Hospital of University of Sao Paulo Medical School, Sao Paulo, Brazil; b Cardiovascular Genetic Department, Heart Institute of Clinical Hospital of University of Sao Paulo Medical School, Sao Paulo, Brazil; c Cardiovascular Surgery Department, Heart Institute of Clinical Hospital of University of Sao Paulo Medical School, Sao Paulo, Brazil; d Cardio-Thoracic Surgery Department, Heart and Vascular Centre, Maastricht University Medical Centre (MUMC) and Cardiovascular Research Centre Maastricht (CARIM), Maastricht, the Netherlands

**Keywords:** heart transplantation, primary graft dysfunction, gene expression, transcriptomics, MYL4

## Abstract

**Background:**

Cardiac transplantation remains the primary treatment for advanced heart failure, with primary graft dysfunction (PGD) being the leading cause of early mortality. While PGD’s exact pathogenesis is unclear, ischemia/reperfusion injury is a key factor. Donor hearts undergo multiple stresses, such as brain death, hypothermia, and ischemia, contributing to PGD and reduced graft survival. Many centers are studying PGD’s pathophysiology through epidemiological, biomarker, and transcriptome analyses. This study aims to evaluate, via transcriptomic analysis of myocardial biopsies, the gene expression profile of patients with PGD and compare it with those without PGD.

**Methods:**

Adult heart transplant patients were included in the study after signing the Informed Consent. The diagnosis of PGD followed the International Society for Heart and Lung Transplant criteria. Clinical and laboratory data from donors and recipients were evaluated. We included 20 consecutive heart transplant patients in the protocol. Gene expression analysis was performed via biopsies at donor heart implantation.

**Results:**

Eleven genes had their expression significantly different in patients with PGD, with MYL4 the less expressed gene, and related to the myosin function and the gene B3GALT 5 the most expressed one and associated with the inflammatory response.

**Conclusions:**

The gene expression in patients with PGD is different when compared to patients without PGD. MYL4 gene was much less expressed in these patients and B3GALT5 was overexpressed. Future studies regarding gene expression on PGD may clarify the pathophysiology and provide possible biomarkers of the disease.

## Graphical abstract

**Figure:**
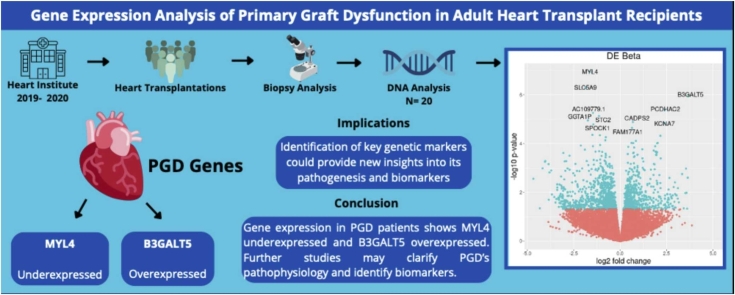

## Background

Heart transplantation remains the preferred treatment for advanced heart failure, despite significant advancements in clinical and surgical care.^1^, ^2^ Globally, an average of 4,000 heart transplants are performed annually, though this likely represents just over half of the actual procedures.^3^ Brazil has emerged as a key player in this field, particularly in treating Chagas disease.^1^ Survival rates have improved dramatically, now averaging 10 years, with some patients surviving over 30 years.^4^, ^5^ These gains are due to factors like refined donor management, organ preservation techniques, and immunosuppressive therapies.^6^, ^7^, ^8^

Despite advances, primary graft dysfunction (PGD) remains the leading cause of early post-transplant mortality. Defined by the International Society for Heart and Lung Transplant (ISHLT) in 2013, PGD involves left, right, or biventricular dysfunction within the first 24 hours post transplant, without other discernible causes.^9^ PGD is multifactorial, influenced by donor and recipient characteristics and the transplant process.^10^, ^11^ Risk factors include ischemia time and the recipient’s systemic inflammatory response, especially in those requiring prolonged mechanical circulatory support.^12^, ^13^, ^14^ The RADIAL score, incorporating both donor and recipient factors, is the only validated tool for predicting PGD.^15^ PGD’s pathogenesis, closely attributed to ischemia/reperfusion injury, starts with the donor and continues post transplant.^16^, ^17^ Genetic studies seek biomarkers and therapeutic targets to mitigate these effects.^18^, ^19^, ^20^, ^21^

Given its prominence, to contribute to a deeper understanding of PGD, the primary objective of this study is to comparatively evaluate the gene expression profile in myocardial tissue samples from transplanted hearts through transcriptomic analysis, focusing on grafts that developed PGD and comparing them with those that did not. Additionally, the study aims to identify the expression levels of key genes involved in the grafts that developed PGD.^22^

## Materials and methods

### Selection criteria

Between June 2019 and December 2020, all adult patients (over 18 years of age) who underwent heart transplantation were invited to participate in the study. The research was approved by the institutional ethics committee (CAAE: 86764218.1.0000.0068), and all patients who agreed signed the Informed Consent Form. The inclusion criterion was patients over 18 years old who underwent heart transplantation at the Heart Institute (InCor-HCFMUSP), and the exclusion criteria were as follows: patients who refused to participate in the study with myocardial dysfunction secondary to hyperacute rejection and with a positive virtual crossmatch, regardless of the value.

### Graft harvesting and myocardial protection

In Brazil, organ donation is legally permitted through family consent. For donation approval, brain death must be diagnosed by 2 qualified physicians who are not part of the transplant teams, in addition to a confirmatory diagnostic test, that is, donor after brain death. The graft harvesting technique followed the same methodology in all cases, with cold static storage. Aortic clamping was performed only after complete cardiac drainage through a wide opening of the inferior vena cava and one of the pulmonary veins. Cardioplegia infusion into the aortic root was then performed, with 2 liters of Custodiol solution infused over 7 to 10 minutes. The temperature of the solution was continuously monitored using a digital thermometer, ideally confirming 4°C. After performing the standard cardiectomy and preparing the graft for transport, an additional 1 liter of the same solution was infused into the aortic root, and the graft was transported to InCor, immersed in Custodiol solution.

### Biopsy collection

**Figure 1:**
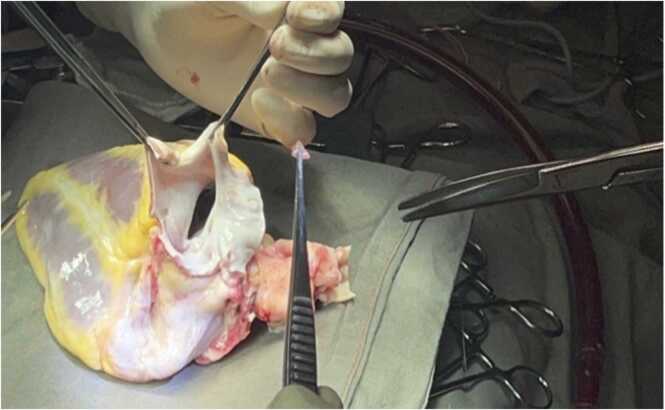
Collection of a left ventricular biopsy, immediately before heart implantation (personal archive).

Endomyocardial biopsies were taken immediately before the start of the heart implantation, already at the transplant center, on back table. Two left ventricular fragments per patient were easily collected, given the wide exposure of the heart chambers (Figure 1). The fragments were stored in RNA-later solution and sent to the InCor genetics laboratory, where they were frozen at −80°C.

### Diagnosis of PGD

PGD was diagnosed based on the criteria established by the ISHLT during the first 24 hours post transplantation. These criteria include left, right, or biventricular dysfunction that is not attributable to other discernible causes such as hyperacute rejection, pulmonary hypertension, or surgical complications. Hemodynamic parameters and echocardiographic findings were used to confirm the diagnosis of PGD, ensuring consistency and accuracy in identifying affected patients.

### Description of the study sample

A total of 40 biopsies from 20 transplants were included at the end of the protocol, comprising the study sample. To characterize the sample, clinical and laboratory data of all recipients were collected, as well as clinical and laboratory data related to the donors and the surgical procedure, as described below.

### Description of recipient data

The following preoperative characteristics were identified in the recipients: age, ethnicity, gender, weight, height, blood type, presence of diabetes mellitus, creatinine levels, hemodynamic data (pulmonary artery systolic pressure and pulmonary vascular resistance), and prioritization type on the transplant list, which could be the use of inotropes, intra-aortic balloon pump, or mechanical ventricular assist device. All recipients included in this sample were prioritized and, therefore, were already hospitalized before transplantation.

The demographic data of the recipients are shown in Table 1. The median age of the patients was 49 years, 55% were male, and 85% were of white ethnicity. Regarding prioritization type, 55% of the patients were prioritized due to the use of an intra-aortic balloon pump, 40% were on inotropes, and 5% were prioritized due to technical chamber reasons. As for the etiology of heart failure, in our study sample, most patients had Chagas disease, followed by idiopathic dilated cardiomyopathy.

**Table 1:** Demographic Characteristics of Heart Transplant Recipients

Recipients	*n* median	% IQR
Median age (IQR)	49	37-55
Median BMI (IQR)	25.4	19.9-28.9
Sex		
Male	11	55
Female	9	45
Ethnicity		
White	17	85
Mixed race	2	10
Black	1	5
Priorization type		
Inotropes	8	40
Intra-aortic balloon pump	11	55
Technical chamber	1	5
Heart failure etiology		
Chagas disease	9	45
Idiopathic dilated cardiomyopathy	6	30
Hypertrophic cardiomyopathy	1	5
Recurrent myxoma	1	5
Valvular disease	1	5
Arrhythmogenic right ventricular dysplasia	1	5

Abbreviations: BMI, body mass index; IQR, interquartile range.

### Description of donor data

Donor data included age, gender, weight, height, ethnicity, blood type, cause of brain death, use of vasoactive drugs, smoking, alcohol, illicit drug use, prior cardiopulmonary arrest, renal function, serum sodium, and echocardiographic data.

The data are presented in Table 2. The majority (75%) were male, with a median age of 21.5 years. The median body mass index was 26.1%, and 65% had blood type O. Traumatic brain injury was the cause of brain death in 70% of the donors, and 30% reported illicit drug use. Additionally, 90% of the donors were on antibiotics. Regarding laboratory tests, the median creatinine level was 1.0, ranging from 0.83 to 1.47. The median ejection fraction of the donors was 66%.

**Table 2:** Characteristics of Donors

Donors characteristics	*n* median	% IQR
Median age (IQR)	21.5	19-35.5
Median BMI (IQR)	26.1	23.7-27.7
Sex		
Male	15	75
Female	5	25
ABO		
A	5	25
B	2	10
O	13	65
Ethnicity		
White	13	65
Mixed race	6	30
Black	1	5
Types of brain death		
Traumatic brain injury (TBI)	14	70
Subarachnoid hemorrhage (SAH)	3	15
Anoxic encephalopathy	2	10
Intracerebral hemorrhage (ICH)	1	5
Systemic arterial hypertension	1	5
Diabetes mellitus	0	0
Alcoholism	2	10
Smoking	5	25
Drug use	6	30
Cardiopulmonary arrest	6	30
Arrest time (min) median (IQR)	8	1-15
Norepinephrine dose (μg/kg/min) median (IQR)	0.145	0.045-0.255
Use of vasopressin	11	55
Use of antibiotics	18	90
Laboratory tests	Mediana	IQR (25-75)
Urea	39.5	28-58
Creatinine	1.01	0.83-1.47
Sodium	149	138.5-160
Echocardiogram		
Left ventricular ejection fraction (%)	66	59-68

Abbreviations: BMI, body mass index; IQR, interquartile range.

### Surgical procedure data

The following data related to the surgical procedure were collected: cardiopulmonary bypass time, total graft ischemia time, need for mechanical circulatory support, and hemodynamic data obtained through the pulmonary artery catheter.

### Gene expression analysis

As previously described, 2 left ventricular fragments were collected for gene expression analysis (transcriptomes). The method for gene analysis is specified below.

### RNA extraction

After homogenizing the tissue and using Qiagen preparation columns, total RNA was extracted. The cardiac muscle tissue samples were homogenized in Trizol (Invitrogen) (1 ml/100 mg of tissue), following the isopropanol and chloroform protocol according to the manufacturer's instructions. Each patient had 2 left ventricular biopsies, which were homogenized together. Once extracted, the RNA was checked for quality and quantity using the Agilent Bioanalyzer and Qubit. Total RNA libraries were prepared using the TruSeq Total RNA kit (Illumina). The libraries were then quantified using Qubit, Bioanalyzer, and Fragment Analyzer equipment, multiplexed, and run on an Illumina HiSeq2500 machine, with approximately 40 million reads per library (biopsy). The resulting fastq files were aligned using standard algorithms (bwa/gtak) with hg19 as the reference genome. For bioinformatics analysis, we initially worked with feature counts and the DESEQ2 package (Differential Expression Analysis for Sequence Count Data), a parametric analysis.

### Gene expression differentiation using the DESEQ2 package

DESEQ2 uses a negative binomial model that includes estimates of gene count dispersion. The parameters of the negative binomial distribution assume that each gene read in RNA sequencing follows a specific pattern, defined by 2 main factors: the mean number of times the gene was read and the gene dispersion factor, which relates to the variability of the analyzed data. Each gene has a characteristic pattern, and this degree of gene dispersion is essential for identifying different expression levels.

The expression level of each gene was demonstrated using *p*-values, adjusted by the magnitude of the difference, expressed as log2 fold change, which ranked the genes according to their expressions, comparing the 2 groups (with and without PGD). Graphical representation was done using 2 methods: comparing fold change with the normal count (MA plots) and comparing the *p*-value with fold change (Volcano plots).

### Statistical analysis

All data were tabulated and statistically analyzed. Continuous data for each variable were compared with the normal curve using the Kolmogorov-Smirnov distance test and classified as parametric and nonparametric. Parametric data were represented by mean and standard deviation of the sample, and independent groups were compared using the Student's *t*-test for nonrepeated measures. Asymmetric data were represented by median and interquartile range (25th and 75th percentiles), and independent groups were compared using the Mann-Whitney test. A significance level of 5% was considered for hypothesis testing. Gene analysis was performed using the software mentioned earlier, specifically designed for RNA sequencing evaluation. The 2 main graphs created from the gene data were the MA and Volcano plots. All statistical analyses were performed using the SPSS statistical package (Software Statistical Package for the Social Science) for Windows, version 13.0, and GraphPad Prism, version 8.

## Results

### Evaluation and post-transplant outcomes

The 20 cases were divided based on the presence or absence of PGD, following the application of ISHLT criteria.

### Perioperative data

Table 3 shows the general characteristics of the patients after being classified according to the presence or absence of PGD. Among the general data, it is noteworthy that all patients who developed PGD had ischemia times of less than 4 hours. The data related to the surgical procedure are shown in Table 4. We identified that the median cardiopulmonary bypass time was 83 minutes, and the median ejection fraction in the first 24 hours was 55%. When separated into groups with and without PGD, patients with PGD showed a lower ejection fraction, as illustrated in Graph 3. Two patients required a new intra-aortic balloon pump, and 4 required circulatory support with extracorporeal membrane oxygenation (ECMO). The first 2 cases involved moderate graft dysfunction, while the latter 4 were cases of severe dysfunction, totaling the 6 PGD cases in the sample.

**Table 3:** General Characteristics of Patients With and Without Primary Graft Dysfunction

	Primary graft dysfunction	
Receptor	Yes (*n* = 6)	No (*n* = 14)
	*n* (%)	*n* (%)
Median age (IQR)	43.5 (29-55)	49 (39-55)
Sex		
Male	4 (66.7)	7 (50.0)
Female	2 (33.3)	7 (50.0)
Ethnicity		
White	5 (83.3)	13 (92.9)
Mixed race	1 (16.7)	0 (0.00)
Black	0 (0.00)	1 (7.1)
Median BMI (IQR)	26.9 (9.8)	25.4 (5.5)
Priorization type		
Inotropes	2 (33.3)	6 (42.9)
Intra-aortic balloon pump	4 (66.7)	7 (50.0)
Technical chamber	0 (0.00)	1 (7.1)
Ischemia time		
<4 h	6 (100)	9 (64.3)
≥4 h	0 (0.00)	5 (35.7)

Abbreviations: BMI, body mass index; IQR, interquartile range.

**Table 4:** Data Related to the Perioperative Period of Heart Transplantation

Recipients parameter	Median	IQR (25-75)
Cardiopulmonary bypass time	83	60.5-120
First 24 h postoperative		
Systolic pulmonary artery pressure (mm Hg)	30	24-43
Central venous pressure (mm Hg)	13.5	10-17.5
Mean arterial pressure (mm Hg)	70	56.5-77.5
Pulmonary capillary wedge pressure (mm Hg)	13.5	12-20
Inotropic score	27.5	6-50
Left ventricular ejection fraction (%)	55	52-70
	*n*	%
Need for mechanical circulatory support		
New IABP	2	10
ECMO	4	20
Primary graft dysfunction		
Moderate	2	10
Severe	4	20

Abbreviations: ECMO, extracorporeal membrane oxygenation; IABP, intra-aortic balloon pump; IQR, interquartile range.

When comparing the hemodynamic data from the first 24 hours, we found that patients with PGD had higher pulmonary pressures and central venous pressure compared to those without PGD, although the differences were not statistically significant. The median inotropic score was 27.5, and when comparing the groups, patients with PGD had a significantly higher score.

### Gene expression analysis

A total of 28.311 transcripts were identified in the cardiac tissue. After quality control measures and expression normalization, 11 transcripts (CADPS2, KCNA7, SPOCK1, FAM177A1, MYL4, SLC5A9, B3GALT5, PCDHAC2, GGTA1P, AC109779.1, and STC2) were found to be associated with the occurrence of PGD. The genes MYL4, SLC5A9, SPOCK1, GGTA1P, AC109779.1, and STC2 were underexpressed, while the genes B3GALT5, PCDHAC2, KCNA7, CADPS2, and FAM177A1 were overexpressed.

**Figure 2:**
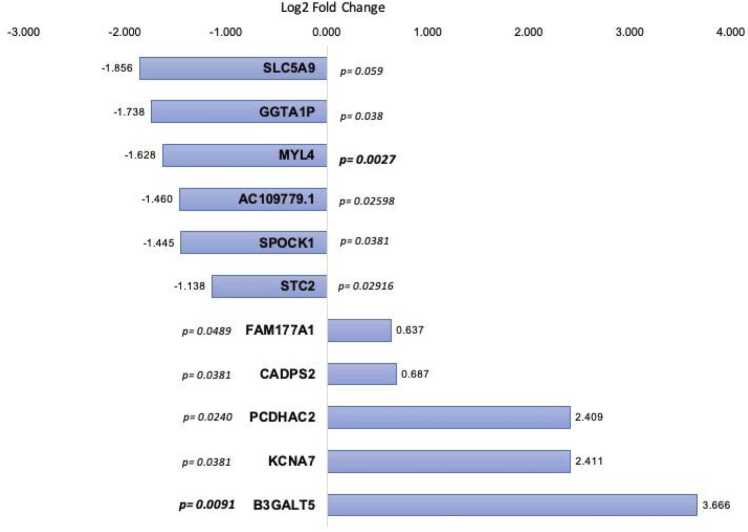
Graph 1—Distribution of differentially expressed genes in hearts with PGD, in a comparative analysis, separated by their expression and *p*-value.

In Graph 1 (Figure 2), the distribution of the 11 differentially expressed genes and their statistical significance are shown. The variable Log2fold change demonstrates the relationship between the degree of gene expression in patients with and without PGD. Negative values indicate lower expression, while positive values indicate higher expression. Notably, the MYL4 gene shows a significantly low expression in PGD patients, while the B3GALT5 gene shows a significantly higher expression in the PGD group.

**Figure 3:**
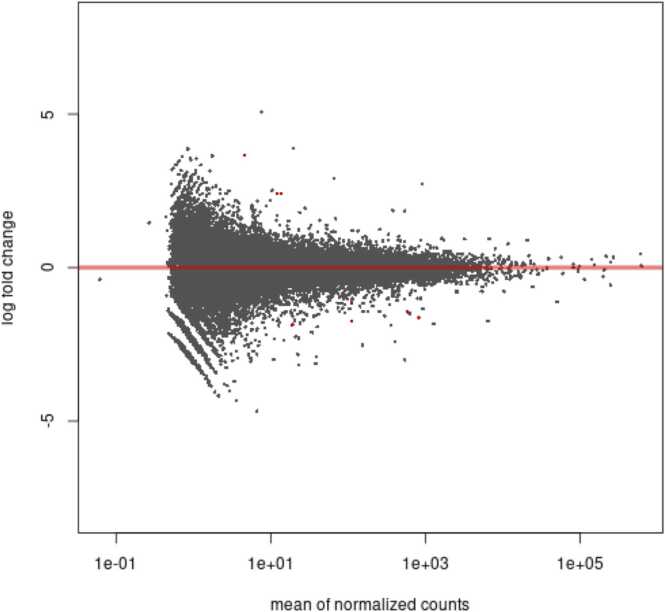
Graph 2—MA plot: distribution of all identified transcripts, highlighting those associated with PGD.

Graph 2 (Figure 3), known as the MA plot, shows the mean expression of all 28,311 transcripts found in the samples, compared with the Log2Fold Change. Thus, the x-axis represents the normal mean expression, and the y-axis shows the magnitude of the difference, represented by the "log2 fold change." The 11 differentially expressed genes are represented by red dots.

**Figure 4:**
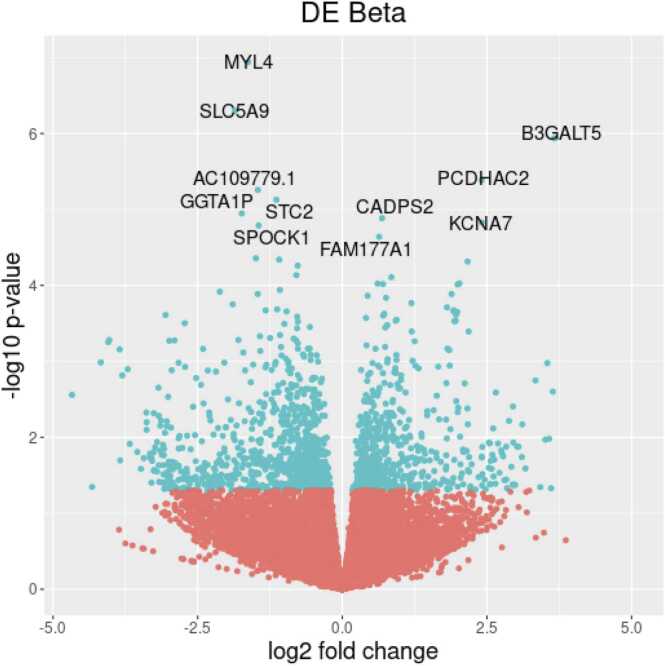
Graph 3—volcano plot" showing genes with higher or lower expression, with statistical significance.

In graph 3 (Figure 4), the "volcano plot", the higher or lower expression of genes and their statistical impact are demonstrated, as the graph combines the *p*-values with the "log2 fold change" values. The underexpressed genes are to the left of the curve, and the overexpressed genes are to the right. The genes that were statistically different in expression in the PGD group are shown at the top of the graph. Two specific genes stand out: MYL4, which was statistically underexpressed, and B3GALT5, which was highly overexpressed.

## Discussion

In this study, we identified eleven genes associated with the development of PGD after heart transplantation using transcriptomic analysis from donor heart tissue obtained immediately before implantation. The most significantly underexpressed gene was myosin light chain 4 (MYL4), while the most overexpressed gene was beta-1,3-Galactosyltransferase 5 (B3GALT5).

MYL4 is part of a large family of motor proteins that share common features, such as ATP hydrolysis (ATPase enzyme activity), actin binding, and the potential for kinetic energy transduction. Originally isolated from muscle cells, myosins are present in nearly all eukaryotic cells. The MYL4 gene encodes an alkali myosin light chain found in embryonic muscle and adult atria. Diseases associated with MYL4 include Atrial Fibrillation and Heart Failure.^23^, ^24^, ^25^, ^26^, ^27^ In a contemporary study, Romero et al. observed several histological changes in hearts explanted due to severe PGD that required retransplants. One of the key findings was the presence of multifocal biventricular ischemic lesions, ranging from early coagulative necrosis to foci of ischemia with neutrophilic inflammation. They also reported diffuse lymphohistiocytic inflammation throughout the myocardium, with a mix of histiocytes, CD3+ T cells, and CD20+ B cells, which was not consistent with acute cellular rejection.^28^ Although the relationship between these histopathological changes and the lower expression of the MYL4 gene remains unclear, future studies focusing on MYL4 could help elucidate the pathophysiology of PGD.

The B3GALT5 gene encodes a member of a family of membrane-bound glycoproteins. The encoded protein may synthesize type 1 Lewis antigens, which are elevated in gastrointestinal and pancreatic cancers. Our findings regarding the overexpression of B3GALT5 could lead to the hypothesis that these encoded proteins might be elevated in donors with a potential risk of PGD. This suggests that the molecular pathways involved in the synthesis of Lewis antigens may play a role in PGD pathogenesis, and further investigation is necessary to explore the utility of B3GALT5 as a biomarker for PGD risk in donor hearts.

Otherwise, the genetic data collected were limited to the donor heart and did not consider interactions between donor and recipient genetic data in our transcriptomic analysis. The hypothesis of this study is that the risk of PGD in the recipient can be detected before transplantation by analyzing these encoded proteins, such as in the donor's blood, as potential biomarkers. Few contemporary studies have investigated potential biomarkers of PGD in heart transplant recipients. For example, one study reported the differential expression of a total of 176 proteins, with pathway analysis suggesting that immune and acute-phase responses, particularly IL-6 signaling, were upregulated in recipients who developed PGD.^29^ However, this study was limited by a very small sample size and, unlike ours, focused on recipient samples rather than donor samples.

Another recently published study by Truby et al. showed that a specific overexpressed gene was related to PGD patients. They analyzed recipient samples at the time of heart transplantation and compared the results with the application of a clinical PGD predictor score (RADIAL score).^30^ While these findings are significant, our study emphasizes the need for further research on donor-specific genetic markers, as our results suggest that PGD may be influenced by factors present in the donor heart before transplantation.

The small number of patients in our cohort is a key limitation of this study, and future studies with larger sample sizes are necessary to confirm our findings. Moreover, the lack of funding is a critical barrier to expanding this type of genetic analysis, which is costly and resource-intensive. Despite these limitations, the study's findings offer important insights into the genetic factors that may contribute to PGD and highlight the potential for incorporating genetic markers like MYL4 and B3GALT5 into pretransplant evaluation protocols.

Looking forward, the clinical utility of PGD risk analysis could be most effectively realized in the outpatient setting as part of an overall pretransplant evaluation. The increasing demand for donors and improved transplant success rates have led to greater utilization of marginal and high-risk donor hearts by many centers. Conducting studies on PGD to offer effective risk analyses for its development is crucial for achieving better post-transplant outcomes. If the risk of PGD could be predicted by analyzing a simple blood sample from the donor, the outcomes of heart transplants could be significantly improved. This would not only allow us to discard potential PGD hearts but also enable the implementation of effective post-transplant management strategies for those identified as high-risk.

### Limitations

This study has several limitations. The small sample size, despite our institution’s ability to perform over 50 transplants annually, limits the generalizability of the findings. This is primarily due to funding constraints, which restricted more extensive genetic analyses. Given the high costs of proteomic and gene expression studies, securing funding is essential for future research. Additionally, we focused solely on donor heart tissue, without considering donor-recipient genetic interactions that could offer a deeper understanding of PGD. The single-center design may also introduce bias, and multicenter studies are needed to validate these findings. The higher incidence of PGD in short-distance retrievals may reflect the small sample size or the inclusion of marginal donors compared to ideal donors in long-distance retrievals. Longer cardiopulmonary bypass time was due to the need for circulatory support (intra-aortic balloon pump,^2^ ECMO^4^) during weaning.

### Future perspectives

Integrating PGD risk analysis into pretransplant evaluations is essential as the use of marginal donors increases. Validating genetic markers like MYL4 and B3GALT5 in larger cohorts and developing noninvasive tests could help predict PGD risk, guiding donor selection and post-transplant strategies to improve recipient outcomes.

## Conclusion

In this study, using transcriptomic analysis of myocardial tissue samples from transplanted hearts, it was observed that tissue gene expression differed in grafts that developed PGD compared to those that did not.

In the grafts that developed PGD, the main genes involved were MYL4, which showed low tissue expression, and B3GALT5, which had increased expression.

## Disclosure statement

This study received no external funding, and the authors have no financial conflicts to declare. All costs were institutionally covered.

We thank the healthcare professionals, transplant teams, and patients for their invaluable contributions.

## References

[1] Bacal F., Silva C.P., Bocchi E.A. (2009). II Diretriz Brasileira de Transplante Cardíaco. Arq Bras Cardiol.

[2] Hunt S.A. (2006). Taking heart: cardiac transplantation past, present, and future. N Engl J Med.

[3] Kfoury A.G., Hammond M.E., Snow G.L. (2012). A longitudinal study of the course of asymptomatic antibody-mediated rejection in heart transplantation. J Heart Lung Transplant.

[4] Taylor D.O., Edwards L.B., Aurora P. (2008). Registry of the International Society for Heart and Lung Transplantation: twenty-fifth official adult heart transplant report-2008. J Heart Lung Transplant.

[5] Khush K.K., Valantine H.A. (2009). New developments in immunosuppressive therapy for heart transplantation. Expert Opin Emerg Drugs.

[6] Sulemanjee N.Z., Merla R., Lick S.D. (2008). The first year post-heart transplantation: use of immunosuppressive drugs and early complications. J Cardiovasc Pharmacol Ther.

[7] Hunt S.A., Haddad F. (2008). The changing face of heart transplantation. J Am Coll Cardiol.

[8] Caves P.K., Stinson E.B., Grahan A.F. (1973). Percutaneous transvenous endomyocardial biopsy. JAMA.

[9] Kobashigawa J., Zuckermann A., Macdonald P. (2014). Report from a consensus conference on primary graft dysfunction after cardiac transplantation. J Heart Lung Transplant.

[10] Russo M.J., Chen J.M., Sorabella R.A. (2010). Factors associated with primary graft failure after heart transplantation. Transplantation.

[11] Ibrahim M., Hendry P., Masters R. (2007). Management of acute severe perioperative failure of cardiac allografts: a single-centre experience with a review of the literature. Can J Cardiol.

[12] Schechter M.A., Watson M.J., Feger B.J. (2016). Elevated cardiac troponin I in preservation solution is associated with primary graft dysfunction. J Card Fail.

[13] George T.J., Arnaoutakis G.J., Beaty C.A. (2012). A novel method of measuring cardiac preservation injury demonstrates University of Wisconsin solution is associated with less ischemic necrosis than Celsior in early cardiac allograft biopsy specimens. J Heart Lung Transplant.

[14] Patarroyo M., Simbaqueba C., Shrestha K. (2012). Pre-operative risk factors and clinical outcomes associated with vasoplegia in recipients of orthotopic heart transplantation in the contemporary era. J Heart Lung Transplant.

[15] Segovia J., Cosio M.D., Barcelo J.M. (2011). RADIAL: a novel primary graft failure risk score in heart transplantation. J Heart Lung Transplant.

[16] Iyer A., Kumarasinghe G., Hicks M. (2011). Primary graft failure after heart transplantation. J Transplant.

[17] Atkinson C., Floerchinger B., Qiao F. (2013). Donor brain death exacerbates complement-dependent ischemia/reperfusion injury in transplanted hearts. Circulation.

[18] Altamirano F., Wang Z.V., Hill J.A. (2015). Cardioprotection in ischaemia-reperfusion injury: novel mechanisms and clinical translation. J Physiol.

[19] Lecour S., Botker H.E., Condorelli G. (2014). ESC working group cellular biology of the heart: position paper: improving the preclinical assessment of novel cardioprotective therapies. Cardiovasc Res.

[20] Raghow R. (2016). An omics perspective on cardiomyopathies and heart failure. Trends Mol Med.

[21] Desai V.G., Fuscoe J.C. (2007). Transcriptional profiling for understanding the basis of mitochondrial involvement in disease and toxicity using the mitochondria-specific MitoChip. Mutat Res.

[22] Nicoara A., Ruffin D., Cooter M. (2017). Primary graft dysfunction after heart transplantation: incidence, trends and associated risk factors. Am J Transplant.

[23] Aharinejad S., Schafer R., Krenn K. (2007). Donor myocardial HIF-1α is an independent predictor of cardiac allograft dysfunction: a 7-year prospective, exploratory study. Am J Transplant.

[24] Marasco S.F., Sheeran F.L., Chaudhuri K. (2014). Molecular markers of programmed cell death in donor heart before transplantation. J Heart Lung Transplant.

[25] Morano M., Zacharzowski U., Maier M. (1996). Regulation of human heart contractility by essential myosin light chain isoforms. J Clin Invest.

[26] Abdelaziz A.I., Pagel I., Schlegel W.P. (2005). Human atrial myosin light chain 1 expression attenuates heart failure. Adv Exp Med Biol.

[27] Abdelaziz A.I., Segaric J., Bartsch H. (2004). Functional characterization of the human atrial essential myosin light chain (hALC-1) in a transgenic rat model. J Mol Med.

[28] Ospina-Romero M., Schulte J.J. (2024). Histopathologic changes of primary graft dysfunction of the heart. J Heart Lung Transplant.

[29] Giangreco N., Lebreton G., Restaino S. (2021). Plasma kallikrein predicts primary graft dysfunction after heart transplant. J Heart Lung Transplant.

[30] Moayedi Y., Truby L.K., Foroutan F. (2024). The International Consortium on Primary Graft Dysfunction: redefining clinical risk factors in the contemporary era of heart transplantation. J Card Fail.

